# Magnetic‐guided catheter ablation of twin AV nodal reentrant tachycardia in a patient with left atrial isomerism, interrupted inferior vena cana, and Kawashima‐Fontan procedure

**DOI:** 10.1002/ccr3.1263

**Published:** 2017-11-13

**Authors:** Francis Bessière, François‐Pierre Mongeon, Judith Therrien, Paul Khairy

**Affiliations:** ^1^ Montreal Heart Institute Université de Montréal Montreal Quebec Canada; ^2^ Jewish General Hospital and MAUDE Unit McGill University Health Centre Montreal Quebec Canada

**Keywords:** Atrial isomerism, catheter ablation, congenital heart disease, magnetic‐guided ablation, twin AV nodes

## Abstract

Twin AV nodal reentrant tachycardia most commonly occurs in patients with complex congenital heart disease who have two distinct AV nodes, His bundles, and non‐preexcited QRS morphologies. Catheter ablation of the weaker AV node may be hindered by anatomical complexities. In such cases, remote magnetic guidance offers a potentially effective solution.

## Introduction

In 1913, Mönckeberg et al. reported the first pathological evidence for the existence of twin atrioventricular (AV) nodes in a patient with AV discordance and a double‐outlet right ventricle 1. A macroreentrant circuit in which both AV nodes are obligatory components, that is, twin AV nodal reentrant tachycardia, has since been described in a few case reports and small case series 2, 3, 4. The most common associated congenital heart defects are AV discordance (with situs solitus or inversus), malaligned complete AV septal defect, and right or left atrial isomerism. Despite anatomical complexities, access for catheter ablation is feasible in most cases by means of a standard femoral venous approach. Herein, we describe successful catheter ablation of twin AV nodal reentrant tachycardia in a patient with no direct access to the heart by inferior or superior veins.

## Case Presentation

A 21‐year‐old man with complex congenital heart disease was referred for catheter ablation after presenting to the emergency room with palpitations and dizziness. A regular narrow complex tachycardia at 176 bpm was terminated upon administration of intravenous adenosine. Over the preceding 2 years, he complained of rapid and regular palpitations of sudden onset that spontaneously resolved within 15 min. He was born with left atrial isomerism (including polysplenia), interruption of the inferior vena cava with azygous vein continuation, bilateral superior vena cavae, double‐outlet right ventricle with a hypoplastic left ventricle, common atrium, complete unbalanced AV septal defect, and D‐malposed great arteries. He underwent a staged Norwood surgical approach, culminating in a Kawashima‐type Fontan. His surgeries included bilateral Glenn shunts (i.e., anastomoses of superior vena cavae to the pulmonary artery), Damus‐Kaye‐Stansel (i.e., anastomosis of the pulmonary artery stump to the aortic root), and a nonfenestrated intracardiac tunnel connecting a confluence of suprahepatic veins to the pulmonary artery. The global ejection fraction of his univentricular heart was 48% by cardiac magnetic resonance (CMR) imaging.

**Figure 1:**
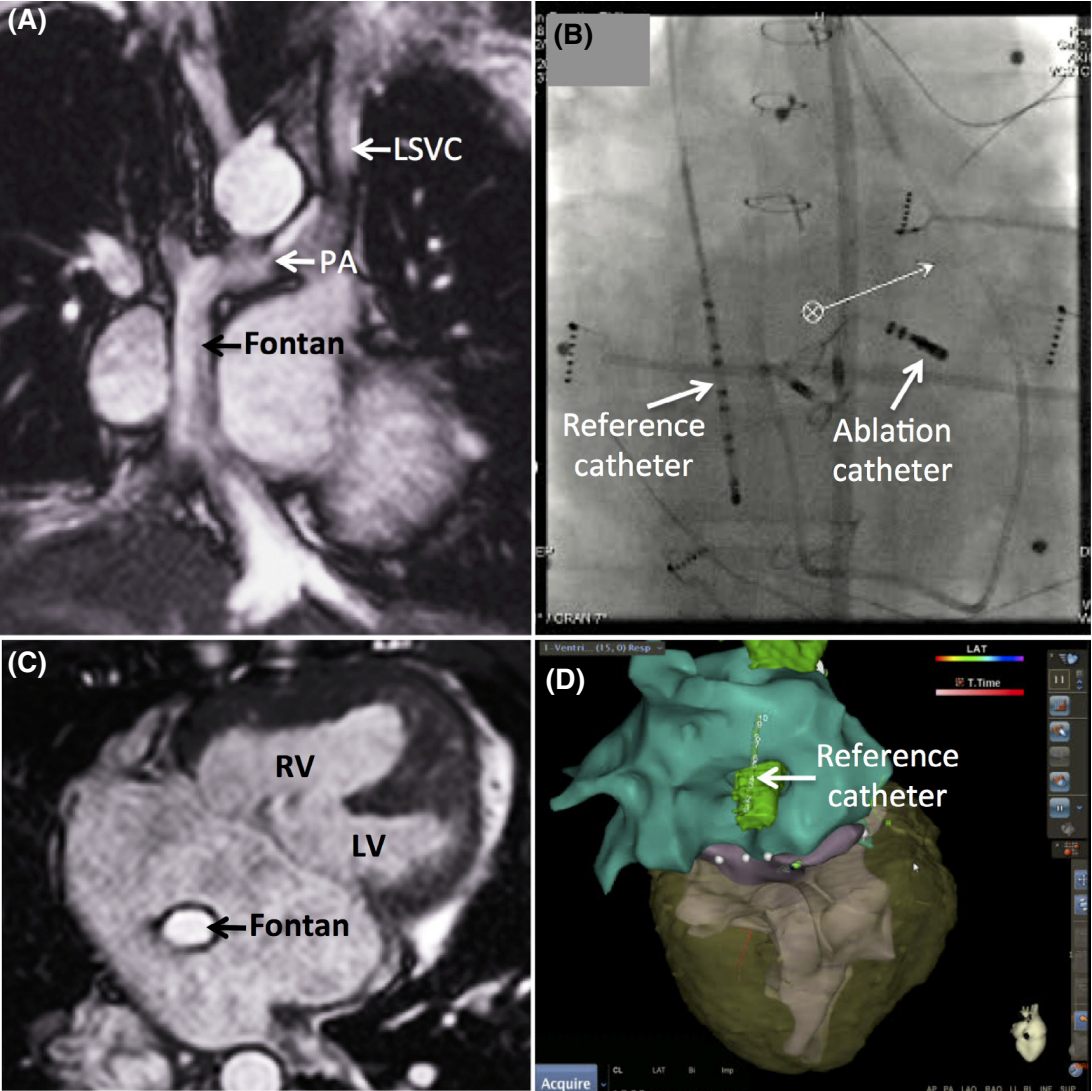
Cardiac anatomy and catheter trajectory. A coronal view of cine‐cardiac magnetic resonance (CMR) imaging is shown in Panel  A. Flow from a confluence of suprahepatic veins is directed to the pulmonary artery (PA) by means of an intracardiac lateral tunnel Fontan variant. Bilateral superior vena cavae are connected to the PA. LSVC denotes left superior vena cava. Panel B displays an anteroposterior fluoroscopic view of the reference catheter placed in the intracardiac lateral tunnel by a right jugular venous approach, and the magnetic‐guided ablation catheter positioned at the superior atrioventricular (AV) node via a retrograde aortic approach. An axial view of cine‐CMR imaging in Panel C shows the intracardiac Fontan in the center of a large common atrium. A common AV valve guards the entrance of a functionally univentricular heart, where right (RV) and left (LV) ventricles communicate through a nonrestrictive bulboventricular foramen. Shown in Panel D is an inferior view of merged electroanatomic mapping with CMR imaging, displaying the position of the reference catheter within the intracardiac Fontan.

Under conscious sedation, an electrophysiological study was performed with three‐dimensional electroanatomic mapping (CARTO 3, Biosense Webster, Johnson & Johnson, South Diamond Bar, CA) and CMR image integration. Considering the lack of venous access, only two catheters were used. A 6‐French decapolar reference catheter (Livewire, St. Jude Medical/Abbott, Menlo Park, CA) was inserted through the right internal jugular vein into the intracardiac tunnel in order to record atrial far‐field signals (Fig. 1). Secondly, an 8‐French radiofrequency ablation catheter (Navistar RMT Thermocool, Biosense Webster) was steered into the heart by remote magnetic guidance (Epoch Solution, Stereotaxis, St. Louis, MO) using a retrograde aortic approach (Fig. 1B).

**Figure 2:**
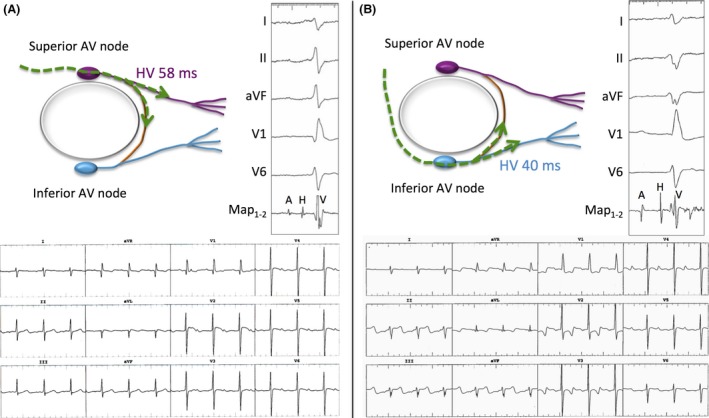
Twin AV nodes. Twin AV nodes are schematically depicted bordering the margins of a complete AV septal defect. Each has its own His‐Purkinje structure, with a so‐called Mönckeberg's sling (orange) of conduction tissue connecting the two systems. Intracardiac and surface ECG recordings in sinus rhythm are shown with electrical conduction coursing along superior (Panel A; purple) and inferior (Panel B; light blue) AV nodes. The top portion of both panels depicts surface ECG recordings from leads I, II, aVF, V1, and V6 along with intracardiac electrocardiograms from the magnetic‐guided ablation catheter positioned to record a His signal. A denotes atrium; H, His; V, ventricle. HV intervals were 58 and 40 msec with conduction across superior and inferior AV nodes, respectively. Twelve‐lead ECGs are shown in the bottom portion of both panels. Note the inferior QRS axis with conduction across the superior AV node, and superior QRS axis with conduction across the inferior AV node.

Electrophysiological testing revealed the presence of two distinct AV nodes, that is, superior and inferior, each with decremental properties, discrete His‐bundle electrograms, and separate patterns of ventricular activation (Fig. 2). The governing rhythm spontaneously transitioned from one AV nodal pathway to the other. The superior AV conduction system was associated with an HV interval of 58 msec, an AV node effective refractory period of 350 msec at a drive train of 600 msec, and an antegrade Wenckebach cycle length of 370 msec. Corresponding values for the inferior AV conduction system were as follows: HV 40 msec, effective refractory period 430 msec, and Wenckebach cycle length 450 msec. A sling of conduction tissue, so‐called Mönckeberg's sling, connected the two AV conduction systems, as schematically portrayed in Figure 2. Signals along the sling were characterized by high‐frequency potentials that preceded local ventricular activation. Conduction across the superior AV node resulted in pre‐excitation of the inferior AV nodal pathway (HV 21 msec) via Mönckeberg's sling. Conduction across the sling was bidirectional, with activation across the inferior AV node associated with pre‐excitation of the superior AV conduction pathway (HV 24 msec).

**Figure 3:**
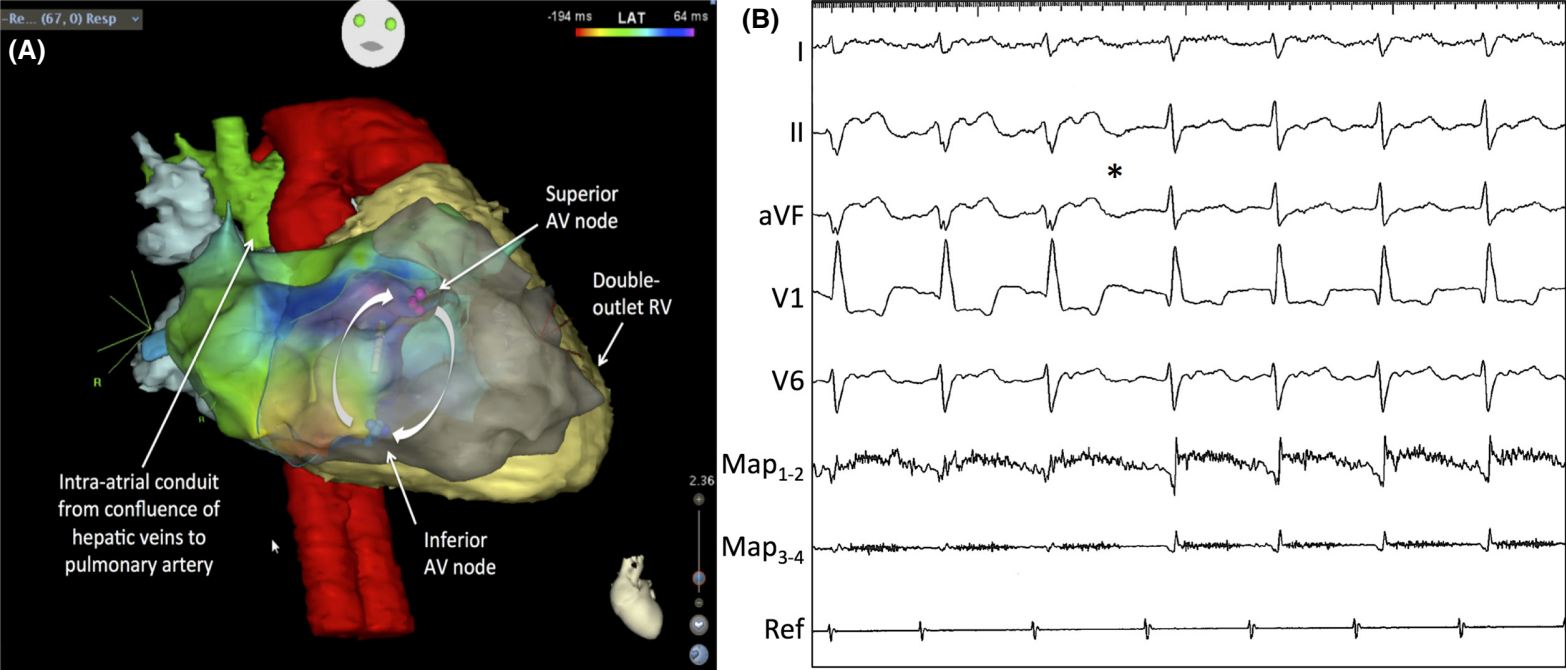
Electroanatomic mapping and catheter ablation. Panel A shows a three‐dimensional electroanatomic map merged with cardiac magnetic resonance imaging. Purple and blue spheres indicate sites with the best His recordings near superior and inferior AV nodes, respectively. Mapping was performed during clinical tachycardia. Local activation times are color‐coded, from red to orange, yellow, green, light blue, dark blue, and purple. A reentrant circuit courses antegrade down the superior AV node, along Mönckeberg's sling, and retrograde across the inferior AV node. The intracardiac Fontan is shown in green, double‐outlet right ventricle (RV) in yellow, and aorta with the pulmonary stump connected to the aortic root (i.e., Damus‐Kaye‐Stansel) in red. Panel B captures surface ECG recordings from leads I, II, aVF, V1, and V6 and intracardiac recordings from the distal (Map_1‐2_) and proximal (Map_3‐4_) magnetic‐guided ablation catheter, and reference (Ref) catheter in the intracardiac conduit. Radiofrequency ablation of the inferior AV node is performed in sinus rhythm during inferior AV conduction. Upon successful ablation, the QRS axis transitions from superior to inferior (asterisk) as conduction shifts from the inferior to the superior AV node.

Sustained antidromic and orthodromic twin AV nodal reentrant tachycardia was induced by atrial extrastimuli under an isoproterenol infusion. During each tachycardia, entrainment mapping at high output from superior and inferior AV nodal pathway sites confirmed that they were both components of the circuit. Electroanatomic mapping of the clinical tachycardia with image integration is shown in Figure 3A. The tachycardia coursed antegrade across the superior AV nodal pathway, down Mönckeberg's sling, and retrograde through the inferior AV node. The inferior AV node was targeted for irrigated radiofrequency ablation considering its less robust conduction properties. As shown in Figure 3B, ablation was performed in sinus rhythm with spontaneous conduction across the inferior AV nodal pathway. Successful elimination of the inferior AV node was marked by a transition of the QRS axis from superior to inferior as conduction shifted to the superior AV nodal pathway. During a 30‐minute observation period on and off isoproterenol, no tachycardia was inducible and no inferior AV node conduction occurred spontaneously or could be provoked by adjacent atrial pacing. At 7 months of follow‐up, the patient remains arrhythmia free with no evidence of inferior AV node conduction.

## Discussion

This case represents a rare description of successful ablation of twin AV nodal reentrant tachycardia using magnetic‐guided ablation in a patient with no direct venous access to the heart. Beyond a retrograde aortic approach, other options to reach the arrhythmia substrate include transhepatic venous access 5 via the confluence of suprahepatic veins and trans‐conduit or transpulmonary puncture from a superior vena cava approach 6. One prior report describes successful magnetic‐guided ablation of twin AV nodal reentrant tachycardia in a 21‐year‐old woman with right atrial isomerism, double‐outlet right ventricle, and an intracardiac total cavopulmonary connection Fontan 7. In this case, access to the intracardiac Fontan was feasible from a left‐sided inferior vena cava.

Characteristic electrophysiological features include (1) two QRS patterns without ventricular pre‐excitation, (2) discrete His bundle electrograms recorded at two separate anatomical locations each associated with a different QRS morphology, (3) decremental conduction over each AV nodal pathway with a stable HV interval, (4) shift to the alternate QRS morphology upon reaching the effective refractory period of the weaker AV nodal pathway, (5) inducible tachycardia with antegrade conduction over one AV nodal pathway and retrograde conduction across the other, (6) entrainment mapping demonstrating that both AV nodal pathways are within the tachycardia circuit, and (7) abolition of the tachycardia circuit by ablation of one AV node. An AV node can be distinguished from a Mahaim fiber by properties such as lack of a delta wave, absence of progressive fusion, typical location, and bidirectional conduction 2.

In addition to the detailed characterization of the rare phenomenon of twin AV nodes, and the connection linking the two conduction systems, the case highlights some of the complexities encountered in catheter ablation procedures in patients with congenital heart disease 8. These may include formidably large chambers of interest, with difficulties ensuring optimal catheter contact and transmural lesions. Distorted anatomies, baffles, conduits, obstructions, and acute angles may impede or preclude access. Occasionally, arrhythmia substrates are concealed beneath patches or prosthetic material. Other challenges include avoidance of inadvertent damage to displaced conduction systems, and complex or multiple arrhythmia circuits. Access by transcatheter punctures across conduits or surgical patches is often performed in patients with complex congenital heart disease but can be prohibitive in certain circumstances.

Magnetic‐guided catheter ablation overcomes some of these challenges and may be particularly well suited for complex anatomies 9. A computer‐controlled system allows the positioning of ablation catheters embedded with magnetic tips. Unlike conventional manual approaches, catheter orientation is controlled at the distal tip. With the Stereotaxis system, the magnetic field is relatively uniform (15 cm diameter), with up to 0.1 Tesla in any direction within the patient's heart. The catheters are soft and flexible, allowing sharp and multiple angle contortions. These features facilitated mapping and ablation using a retrograde aortic approach. It is theoretically possible that the substrate targeted for ablation, that is, inferior AV node, could be reached by a conventional retrograde approach. However, it is uncertain whether stability and contact would be sufficient for successful ablation. Moreover, the anatomical complexities render it unlikely that comprehensive three‐dimensional electroanatomic mapping could be achieved with manual catheter manipulation. In addition, the associated radiation exposure time would likely greatly exceed the 4.5 min attained in our patient.

Additional features of magnetic‐guided ablation particularly suited for complex substrates include integration with three‐dimensional electroanatomic mapping systems, computer automation, coregistration of preprocedural images, and advanced tools to enable more intuitive navigation, storage, and data sharing. Irrigated radiofrequency catheter ablation technology was integrated in 2008, permitting the creation of larger and deeper lesions. Potential drawbacks include the relatively stiff catheter tip embedded with magnetic rings that may impede direct access to certain areas and lack of real‐time contact force monitoring. Plans are underway to create an international registry to systematically assess safety and efficacy of magnetic‐guided catheter ablation in patients with congenital heart disease via the Pediatric and Congenital Electrophysiology Society (PACES).

## Conclusion

In conclusion, twin AV nodal reentrant tachycardia is a rare supraventricular arrhythmia that most commonly occurs in patients with combinations of AV discordance, malaligned AV septal defect, and atrial isomerism. As this case demonstrates, characteristic electrophysiological features include two distinct AV nodes with discrete His bundle electrograms, decremental conduction, and separate non‐preexcited QRS morphologies; a sling of tissue (Mönckeberg's sling) connecting the two AV conduction systems; and tachycardia that courses antegrade by one AV nodal pathway, across Mönckeberg's sling, and retrograde via the second AV nodal pathway. Catheter ablation, which consists of targeting the weaker AV node, may be hindered by anatomical complexities including lack of transvenous access. Magnetic‐guided ablation offers a potential solution by permitting a soft flexible ablation catheter to be steered into the heart by a retrograde aortic approach. Safety and efficacy of magnetic‐guided ablation in patients with congenital heart disease remain to be assessed on a large scale.

## Conflict of Interest

None declared.

## Authorship

FB: Dr. Bessière acquired electrophysiological data and drafted the manuscript. F‐PM: Dr. Mongeon acquired imaging data and made critical revisions to the manuscript. JT: Dr. Therrien acquired clinical data and made critical revisions to the manuscript. PK: Dr. Khairy acquired the data, drafted the manuscript, and handled supervision.
